# Supplementary figures and images for: Mushroom body subsets encode CREB2-dependent water-reward long-term memory in Drosophila
Source: PLoS Genet. 2020 Aug 11;16(8):e1008963. doi: 10.1371/journal.pgen.1008963 (PMC7418956; doi:10.1371/journal.pgen.1008963)

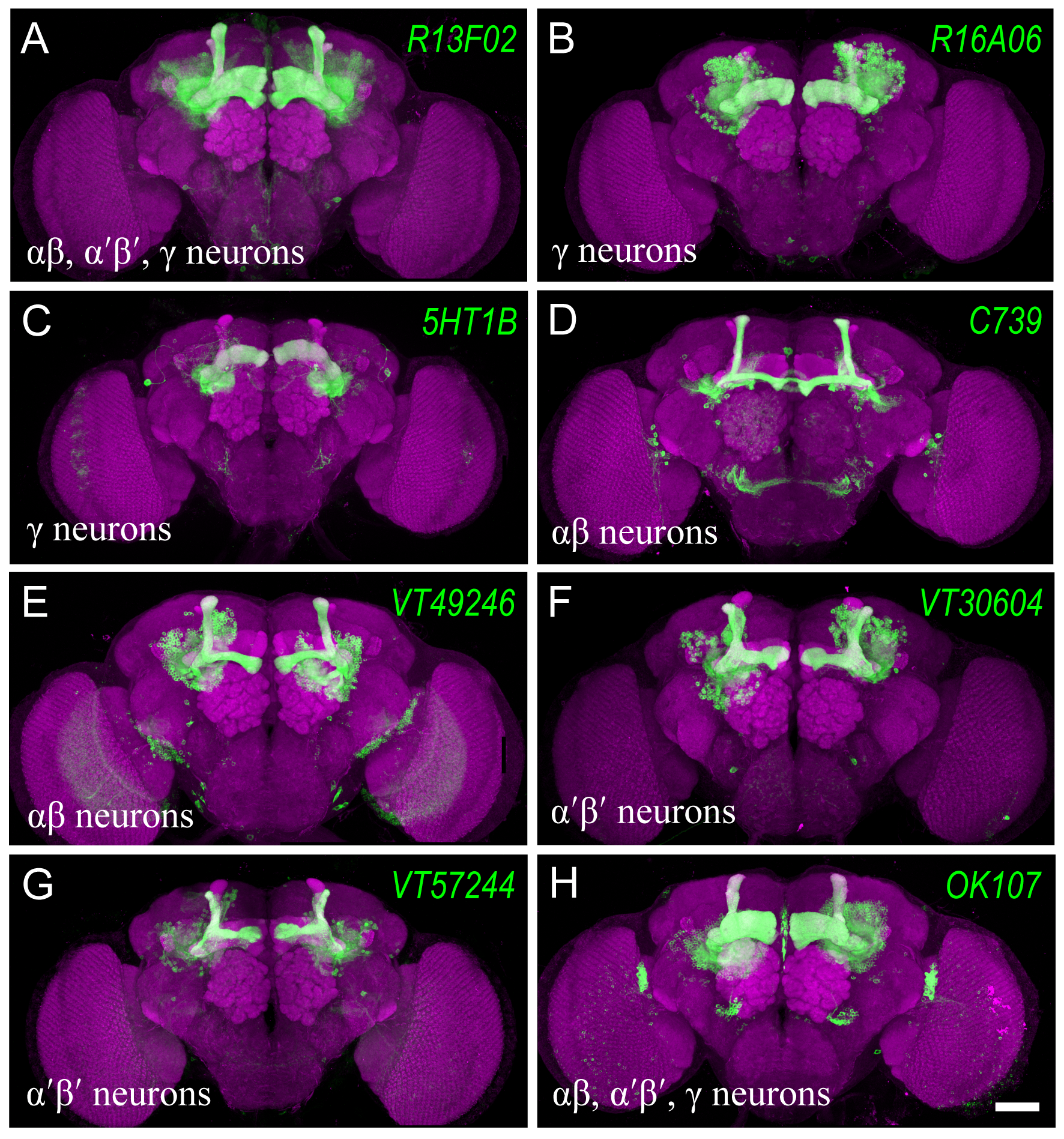

Supplement: S1 Fig — (A) The expression pattern of R13F02-GAL4-driven GFP expression in γ, αβ, and α′β′ neurons. (B) The expression pattern of R16A06-GAL4-driven GFP expression in γ neurons. (C) The expression pattern of 5HT1B-GAL4-driven GFP expression in γ neurons. (D) The expression pattern of C739-GAL4-driven GFP expression in αβ neurons. (E) The expression pattern of VT49246-GAL4-driven GFP expression in αβ neurons. (F) The expression pattern of VT30604-GAL4-driven GFP expression in α′β′ neurons. (G) The expression pattern of VT57244-GAL4-driven GFP expression in α′β′ neurons. (H) The expression pattern of OK107-GAL4-driven GFP expression in γ, αβ, and α′β′ neurons. The brain neuropils were immunostained with anti-DLG antibody (magenta). Scale bar represents 50 μm. (TIF) [file pgen.1008963.s001.tif]

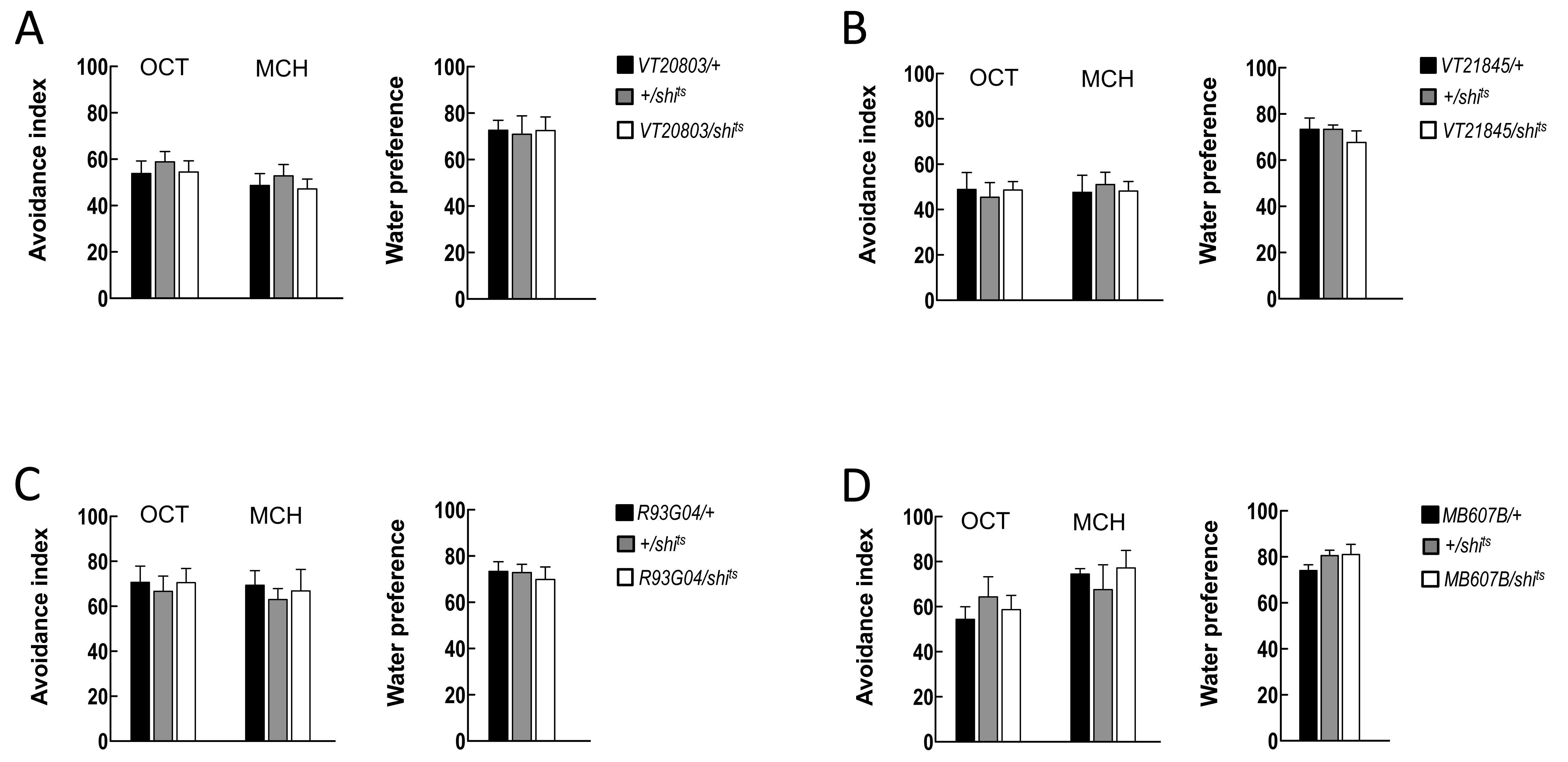

Supplement: S2 Fig — (A) Normal olfactory acuity to OCT or MCH and normal water preference at restrictive temperature (32°C) in thirsty VT20803-GAL4 > UAS-shits flies. Each value represents mean ± SEM, (N = 14 for olfactory acuity; N = 8 for water preference). p > 0.05; one-way ANOVA. (B) Normal olfactory acuity to OCT or MCH and normal water preference at restrictive temperature (32°C) in thirsty VT21845-GAL4 > UAS-shits flies. Each value represents mean ± SEM, (N = 6 for olfactory acuity; N = 6 for water preference). p > 0.05; one-way ANOVA. (C) Normal olfactory acuity to OCT or MCH and normal water preference at restrictive temperature (32°C) in thirsty R93G04-GAL4 > UAS-shits flies. Each value represents mean ± SEM, (N = 6 for olfactory acuity; N = 6 for water preference). p > 0.05; one-way ANOVA. (D) Normal olfactory acuity to OCT or MCH and normal water preference at restrictive temperature (32°C) in thirsty MB607B-GAL4 > UAS-shits flies. Each value represents mean ± SEM, (N = 6~8 for olfactory acuity; N = 8~9 for water preference). p > 0.05; one-way ANOVA. (TIF) [file pgen.1008963.s002.tif]

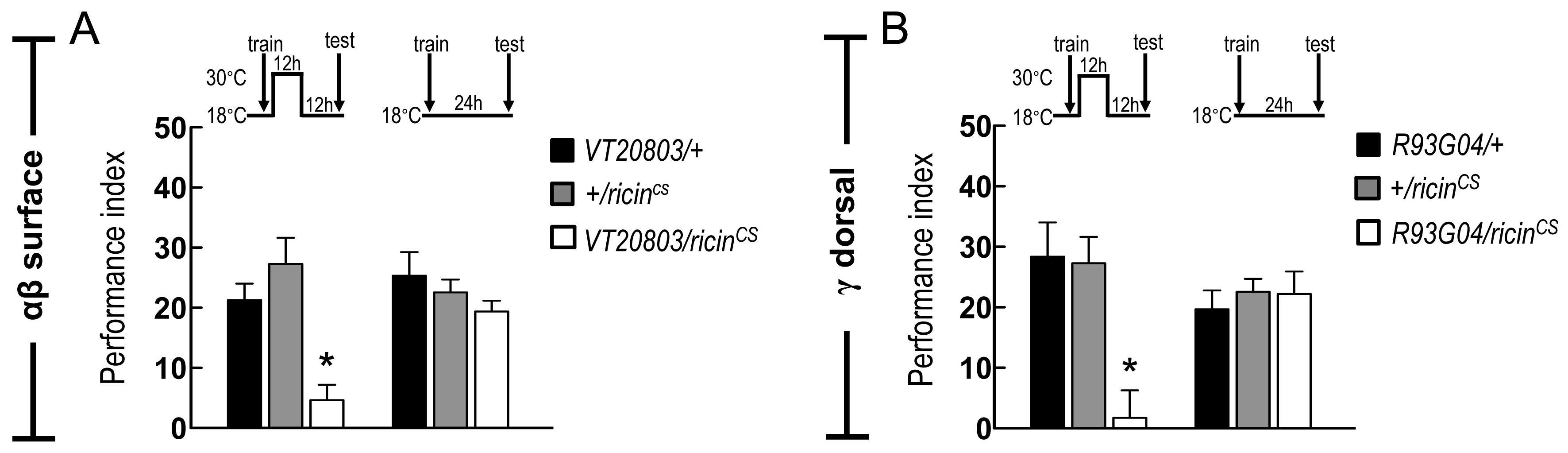

Supplement: S3 Fig — (A) Blocking protein synthesis in αβ surface neurons using VT20803-GAL4 to drive the expression of activated RICINCS (30°C) during memory formation impaired wLTM (left panel). Each value represents mean ± SEM (N = 11). *, p < 0.05; one-way ANOVA followed by Tukey’s test. The 24-hour water-reward memory was normal with inactive RICINCS (18°C) expression in αβ surface neurons all the way during behavioral assay (right panel). Each value represents mean ± SEM (N = 8~9). p > 0.05; one-way ANOVA. (B) Blocking protein synthesis in γ dorsal neurons using R93G04-GAL4 to drive the expression of activated RICINCS (30°C) during memory formation impaired wLTM (left panel). Each value represents mean ± SEM (N = 11). *, p < 0.05; one-way ANOVA followed by Tukey’s test. The 24-hour water-reward memory was normal with inactive RICINCS (18°C) expression in γ dorsal neurons all the way during behavioral assay (right panel). Each value represents mean ± SEM (N = 9~10). p > 0.05; one-way ANOVA. (TIF) [file pgen.1008963.s003.tif]

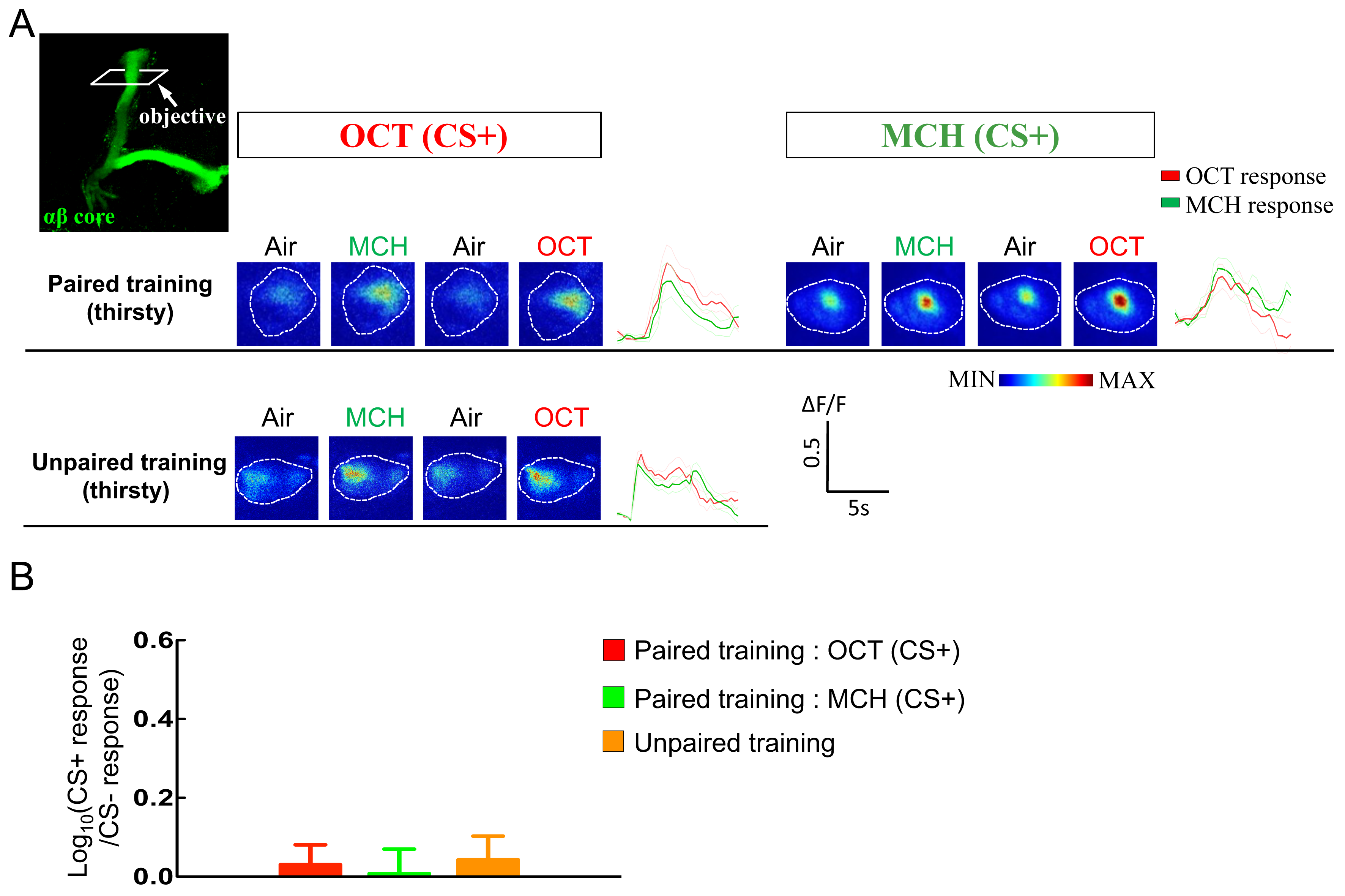

Supplement: S4 Fig — (A) The GCaMP6 response 24-hour after water-reward conditioning was assayed in αβ core neurons (the image-recording region is showed in the top left figure). For the paired training group: flies received CS− odor without water-reward (US), followed by exposure to the CS+ odor with water-reward. For the unpaired training group: flies received CS− odor without water-reward, followed by exposure to CS+ odor without water-reward, and the water-reward was delivered 1-minute later after CS+ odor. Odor/water paired training did not induce wLTM trace 24-hour post-conditioning in the α-lobe region of the αβ core neurons to the training odor [OCT-trained flies: OCT (CS+), MCH-trained flies: MCH (CS+)] in thirsty-state. (B) Quantification of the GCaMP6 responses to the training odor (CS+) relative to the non-training odor (CS−) in the α-lobe region of αβ core neurons 24-hour post-conditioning in OCT-trained (red bar) or MCH-trained (green bar) flies. The Log ratios of the CS+ response to the CS− response were calculated using the peak response amplitudes. Each value represents mean ± SEM (N = 6~11). Each bar is not statistically significantly different from zero, p > 0.05; one sample t-test. Genotype: UAS-GCaMP6m/+; VT0841-GAL4/+. (TIF) [file pgen.1008963.s004.tif]

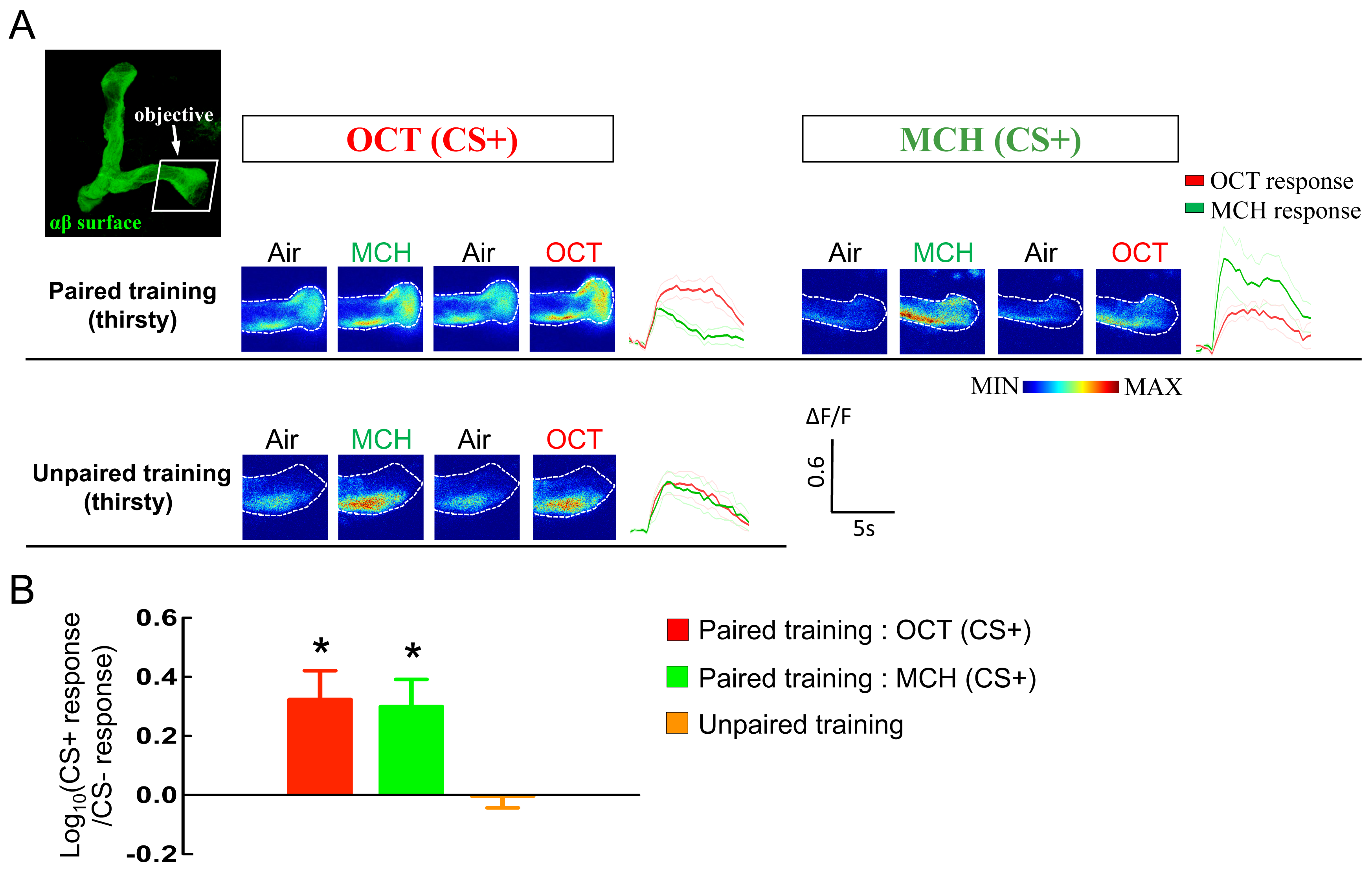

Supplement: S5 Fig — (A) The GCaMP6 response 24-hour after water-reward conditioning was assayed in αβ surface neurons (the image-recording region is showed in the top left figure). For the paired training group: flies received CS− odor without water-reward (US), followed by exposure to the CS+ odor with water-reward. For the unpaired training group: flies received CS− odor without water-reward, followed by exposure to CS+ odor without water-reward, and the water-reward was delivered 1-minute later after CS+ odor. Odor/water paired training induced an increase in the GCaMP6 responses in the β-lobe region of the αβ surface neurons to the training odor [OCT-trained flies: OCT (CS+), MCH-trained flies: MCH (CS+)] in thirsty-state. (B) Quantification of the increased GCaMP6 responses to the training odor (CS+) relative to the non-training odor (CS−) in the β-lobe region of αβ surface neurons 24-hour post-conditioning in OCT-trained (red bar) or MCH-trained (green bar) flies. The Log ratios of the CS+ response to the CS− response were calculated using the peak response amplitudes. Each value represents mean ± SEM (N = 6~8). *, p < 0.05; statistically significantly different from zero; one sample t-test. Genotype: UAS-GCaMP6m/+; VT20803-GAL4/+. (TIF) [file pgen.1008963.s005.tif]

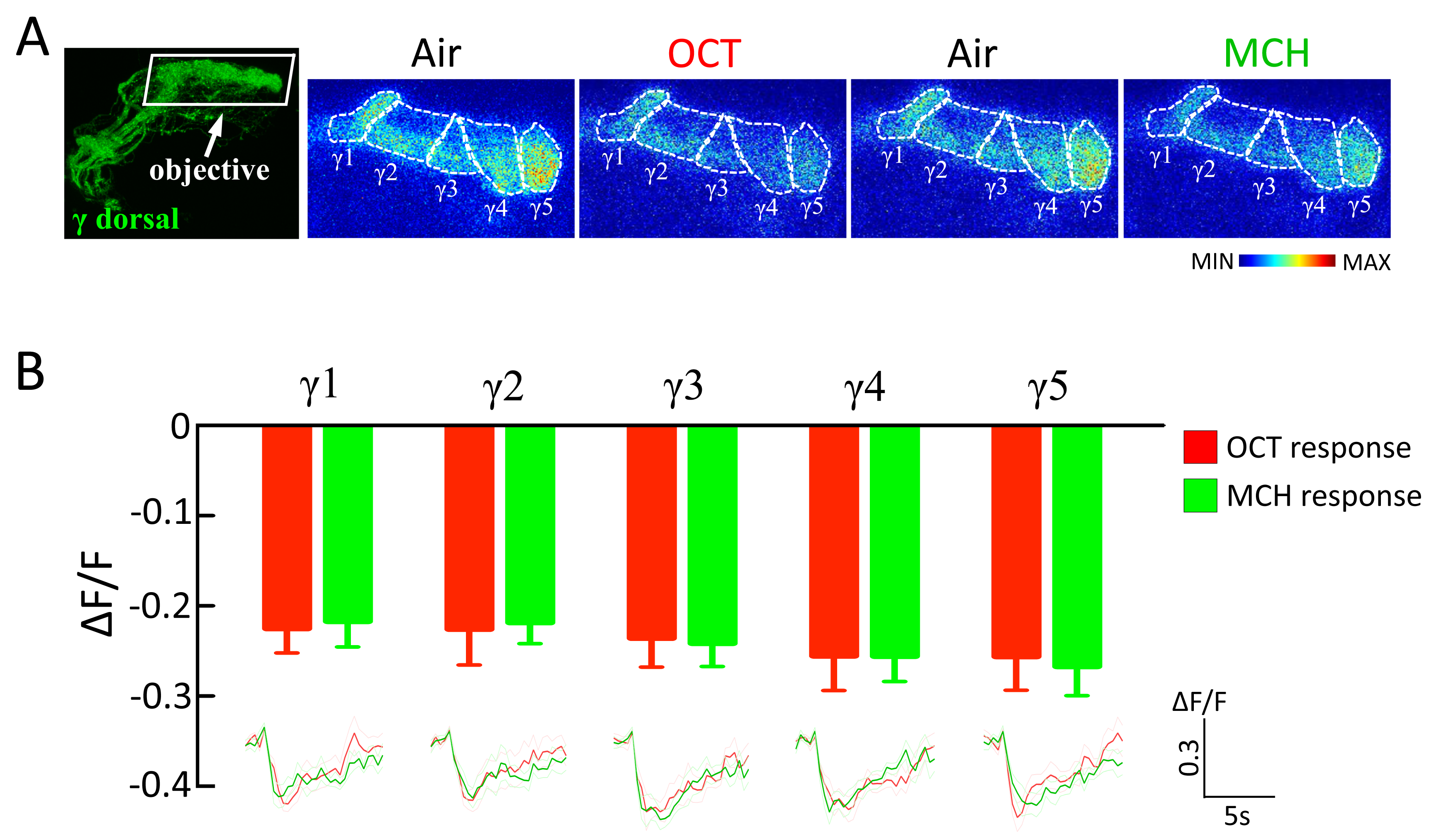

Supplement: S6 Fig — (A) Naïve flies carrying UAS-GCaMP6m/+; R93G04-GAL4/+ transgenes were used to perform calcium imaging experiment of odor response. Flies show significantly decreased calcium responses to OCT and MCH in each γ dorsal subdomain. (B) Quantification of the GCaMP6 responses to OCT and MCH in each γ dorsal subdomain in naïve flies. Each value represents mean ± SEM (N = 9). (TIF) [file pgen.1008963.s006.tif]

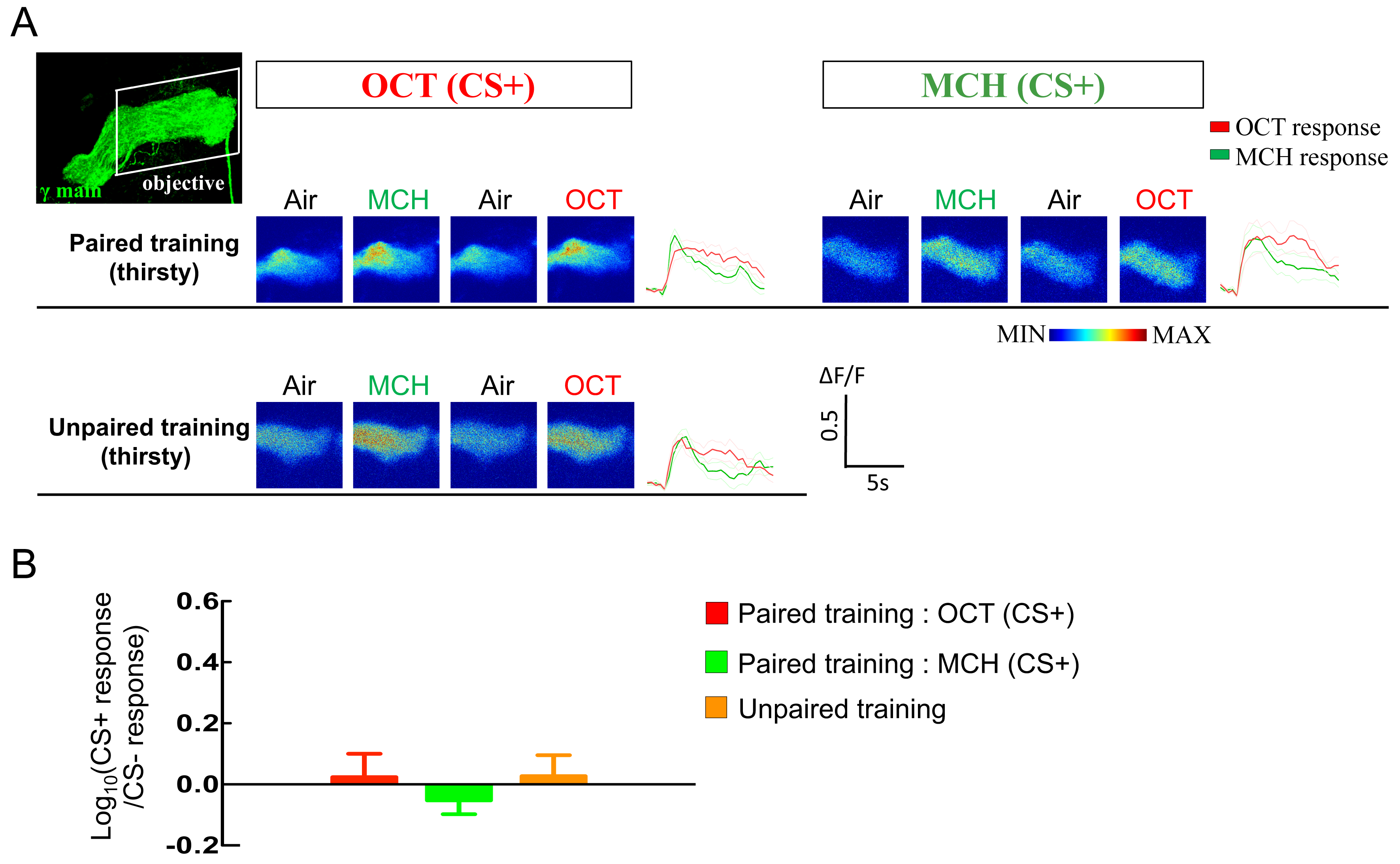

Supplement: S7 Fig — (A) The GCaMP6 response 24-hour after water-reward conditioning was assayed in γ main neurons (the image-recording region is showed in the top left figure). For the paired training group: flies received CS− odor without water-reward (US), followed by exposure to the CS+ odor with water-reward. For the unpaired training group: flies received CS− odor without water-reward, followed by exposure to CS+ odor without water-reward, and the water-reward was delivered 1-minute later after CS+ odor. Odor/water paired training did not induce wLTM trace 24-hour post-conditioning in the γ-lobe region of the γ main neurons to the training odor [OCT-trained flies: OCT (CS+), MCH-trained flies: MCH (CS+)] in thirsty-state. (B) Quantification of the GCaMP6 responses to the training odor (CS+) relative to the non-training odor (CS−) in the γ-lobe region of the γ main neurons in OCT-trained (red bar) or MCH-trained (green bar) flies 24-hour post-conditioning. The Log ratios of the CS+ response to the CS− response were calculated using the peak response amplitudes. Each value represents mean ± SEM (N = 7). Each bar is not statistically significantly different from zero, p > 0.05; one sample t-test. Genotype: UAS-GCaMP6m/+; R64C08-GAL4/+. (TIF) [file pgen.1008963.s007.tif]
