# Supplementary material for: Mushroom body subsets encode CREB2-dependent water-reward long-term memory in Drosophila
Source: PLoS Genet. 2020 Aug 11;16(8):e1008963. doi: 10.1371/journal.pgen.1008963 (PMC7418956; doi:10.1371/journal.pgen.1008963)
Supplement: Attachment — Submitted filename: Lee_Response to Reviewers.docx [file pgen.1008963.s009.docx]

Reviewer #1: This work systematically investigates the location of water-reward long-term memory (wLTM) among different types of the mushroom body (MB) neurons in Drosophila. The data are very solid. The results are instructive for studying neural circuits of long-term memory. It is suitable to publish in PLOS Genetics. There are some minor concerns below.

1, In the abstract, “protein synthesis followed by the conserved cAMP response element binding protein (CREB)-dependent gene transcription”. The order of protein synthesis and transcription seems reversed.

Author’s reply:

Thank you for your helpful comment. We have revised the sentence to “Long-term memory (LTM) formation depends on the conserved cAMP response element-binding protein (CREB)-dependent gene transcription followed by *de novo* protein synthesis”. Page 1, lines 21-23.

2, Line 49, “… required for retrieval, but not for acquisition or consolidation of wLTM”. There is no evidence for such a statement in this manuscript. The previous study should be cited here.

Author’s reply:

We have added the related reference (Shyu WH et al., 2017 *Nat. Comm.*) as suggested.

3, In figure 2 and figure 5, it is more clear and helpful for readers if the information about neuron types (γ main, γ dorsal, or …) driven by different Gal4 lines is also provided in the figures.

Author’s reply:

We have added the information regarding each mushroom body neuron types in Fig 2 and Fig 5 as suggested.

4, In figure 6 and figure 7, it is better to provide a sketch of imaging protocol (time points for thirsty, training, imaging, and how to perform unpaired training).
Author’s reply:

We have added a diagram illustrating the protocols of paired training, unpaired training, and *in vivo* calcium imaging in Fig 6 and Fig 7 as suggested.

Reviewer #2: In this manuscript, Lee et al characterize the role of the mushroom bodies in forming long-term water memories (wLTM). Drosophila have long been a leading model for the study of associative memory formation, historically relying on a paradigm where electric shock is paired with an odor. The study of water memories provide a number of significant advantages, including its ethological relevance and greater potential to map neural circuits carrying the US to the mushroom bodies. Therefore, studying wLTM is likely to be of broad interest to the community. This manuscript describes an acute requirement for protein synthesis in the mushroom bodies, and identifies physiological changes in defined lobes that are associated with memory. Overall, the manuscript is well written and scientifically sound. I have some concerns about whether the findings presented represent a significant advance over the groups 2017 paper that defined MB-neurons required for LTM formation. I have made a number of suggestions below that may help address potential weaknesses and enhance the impact of the manuscript. Overall, this manuscript is likely to be well received based on the under-studied nature of wLTM and the identification of physiological changes within specific MB lobes that associate with memory formation.

Major comments
1. 2.Based on the temperature shift protocols, it seems that expression dCREB2 and dRicin for 8 hrs after training impairs long-term memory. An alternative explanation is these manipulations induce irrevocable harm to the neurons. For these reasons the conclusion really requires a time-point the flies can recover from, and not be impacted (for example, 8-16hrs after training, or immediately prior to training).

Author’s reply:

For the *dCREB2b* experiments:

First, our *in vivo* calcium imaging data indicated that the mushroom body neurons show normal odor responses to OCT and MCH (Fig 6 and Fig 7) in adult stage-specific expression of *dCREB2b* transgene by using the same temperature shifting protocol, which suggest this manipulation did not induce the irrevocable harm to mushroom body neurons. Second, it has been shown that fly has normal mushroom body growth morphology with adult-stage-specific expression of *dCREB2b* transgene (using *tub-GAL80^ts^* inhibition with 3-day or 7-day temperature shifting protocol, see below **Figure R1, G-I**; image is adapted from Chen CC et al., 2012 *Science*). However, constitutive expression of *dCREB2b* in mushroom body throughout the development indeed causes the developmental defect in mushroom body neurons (see below **Figure R1, D-F**; image is adapted from Chen CC et al., 2012 *Science*). Taken together, the mushroom body neurons show normal growth morphology and normal odor responses with inducible expression of *dCREB2b* by *tub-GAL80^ts^* in adult flies, which suggest this genetic manipulation does not induce irrevocable harm to mushroom body neurons.

**Figure R1.** (**D-F)** Constitutive expression of CREB2 repressor produces morphological defects in MB neurons. Arrow indicates missing α lobes; dashed circle indicates fewer fibers in γ lobes; arrowhead indicates some αβ axons crossing the midline. (**G-I**) Induced expression of CREB2 repressor in MB neurons does not growth morphology of MB neurons. Flies were raised and then at 18°C were transferred to 30°C for at least 3 days before training began, to remove *tub-Gal80^ts^* inhibition and allow *Gal4*-mediated transgene expression (adapted from Chen CC et al., 2012 *Science*, Fig S10).

For Ricin^cs^ experiments:

It has been shown that inhibition of protein synthesis in *per-GAL4* expression neurons via activated RICIN^CS^ disrupts 24-hour aversive memory after spaced training (see below **Figure R2**; image is adapted from Chen CC et al., 2012 *Science*). If activated RICIN^CS^was restricted to the first 12 hours after training, 24-hour aversive memory after spaced training was impaired. However, if activated RICIN^CS^ was restricted to the last 12 hours after training, 24-hour aversive memory after spaced training was normal (see below **Figure R2, right panel**; image is adapted from Chen CC et al., 2012 *Science*). These results suggest the 12 hours temperature shift in *ricin^CS^*-related experiments did not cause irrevocable harm to the neurons. Otherwise, the memory defect should been observed in the *per-Gal4 > UAS-ricin^CS^* *(per/ricin^CS^)* fly group with heat shock at the last 12 hours after training.

**Figure R2. (D)** Inhibition of protein synthesis in *per-Gal4* neurons at different time windows after training impairs 1-day memory after spaced training but not after massed training. 1-day memory after spaced training is normal when RICIN^CS^ is inactive (in flies kept at 18 °C). 1-day memory after spaced, but not massed, training was impaired, when RICIN^CS^ was activated (flies were transferred to 30 °C) immediately after training for the entire 24 hours. If activated RICIN^CS^ was restricted to the first 12 hours after training, 1-day memory after spaced training again was impaired. If activated RICIN^CS^ was restricted to the last 12 hours after training, however, 1-day memory after spaced training was normal. Values are means + S.E.M. (N = 8-12 experiments; *, *P* < 0.05; ***, *P* < 0.001). Genotypes: 1) *+*/*+*, 2) *per-Gal4*/*+*, 3) *UAS-ricin^CS^*/*+* and 4) *per-Gal4*/*+*;*UAS-ricin^CS^*/+. (adapted from Chen CC et al., 2012 *Science*, Fig S4D).

2. For shibire experiments in figure 3, it would be helpful to also examine the consolidation period after testing, when protein-synthesis is found to be critical.

Author’s reply:

In our previous published work, we blocked the synaptic output via *shibire^ts^* in mushroom body αβ neurons by *VT49246-GAL4* or *C739-GAL4* during different periods of wLTM formation, and found that synaptic output of mushroom body αβ neurons is only required for wLTM retrieval but not for acquisition and consolidation (see below **Figure R3**; image is adapted from Shyu WH et al., 2017 *Nat. Comm.*). The expressions of *VT49246-GAL4* and *C739-GAL4* include αβ core, αβ surface, and αβ posterior neuron subsets. Therefore, in the current study, we only behaviorally examined the wLTM retrieval period via *shibire^ts^* blockage in αβ core, αβ surface, or αβ posterior neuron subset, respectively.

**Figure R3. (e)** Blocking the output of αβ neurons (*VT49246-GAL4*) using *shi^ts^* during retrieval but not during acquisition and consolidation, impaired LTM. Each value represents mean ± s.e.m. (N = 8 for each bar). *P < 0.05; ANOVA followed by Tukey’s test. **(f)** Blocking the output of αβ neurons (*C739-GAL4*) using *shi^ts^* during retrieval but not during acquisition and consolidation, impaired LTM. Each value represents mean ± s.e.m. (N = 8 for each bar). *P < 0.05; ANOVA followed by Tukey’s test. (adapted from Shyu WH et al., 2017 *Nat. Comm.*, Fig 6).

3. I have concerns about the use of the word engram throughout the manuscript. My understand is engram is the site of memory formation. Here, it seems this is interpreted more broadly and applied to any physiological change associated with memory.

Author’s reply:

Thank you for your comments, we have revised all the descriptions of “engram” in whole manuscript as suggested.

4. Discussion does nto sufficiently address a few key aspects that will represent these findings in the broader context of fly memory. First, many types of fly memories are mushroom body dependent. At the level of neural circuits, what is shared and what is different?

Author’s reply:

We have added a paragraph in the Discussion section for comparing other types of fly olfactory memory with our current findings, in neural circuit levels. Page 10, lines 232-255. See below:

“Neurotransmission from αβ neurons is required for both shock-punitive and sugar-reward LTMs retrieval [28], whereas neurotransmission from γ neurons is dispensable for retrieval of both shock-punitive and sugar-reward LTMs [28, 29]. However, our previous study showed that the neurotransmission from αβ and γ neurons is required for wLTM retrieval [1] suggesting that wLTM is different from the other types of olfactory associative LTMs at MB circuit levels. The αβ and γ neurons are classified into αβ core, αβ surface, αβ posterior, γ main, and γ dorsal neuron subsets according to the morphology of their axons [15]. It has been shown that neurotransmission from the combination of MB αβ surface and αβ posterior subsets is necessary for the retrieval of both shock-punitive and sugar-rewarded LTMs [30]. Another study suggests that the neurotransmission from αβ surface and αβ core subsets is required for the retrieval of sugar-reward LTM [31]. These results imply that several different subsets of αβ neurons participate in the retrieval of *Drosophila* sugar-reward LTM. Herein, we showed that neurotransmission only from αβ surface is necessary for wLTM retrieval, whereas the αβ core and αβ posterior subdivisions are dispensable (Fig 3 and S2 Fig). Contrary to the sugar reward conditioning in which γ neurons are dispensable for the retrieval of sugar-reward LTM [28, 29], wLTM retrieval requires neurotransmission from γ dorsal but not from γ main neuron subset (Fig 4 and S2 Fig). Taken together, these results imply that γ dorsal neuronal activity is specifically required for wLTM retrieval but not for sugar-reward LTM [28, 29]. Adult stage-specific expression of *dCREB2b* or blocking *de novo* protein synthesis in αβ surface and γ dorsal neurons disrupts wLTM, further suggesting the crucial role of αβ surface and γ dorsal neurons in *Drosophila* wLTM process (Fig 5 and S3 Fig).”

Second, are there further subsets of neurons within the MBs, and if so, how should this be interpreted given that the driver lines used for imaging cannot parse these subsets?
Author’s reply:

To the best of our knowledge, there are only seven different cell types of MB neurons. By using the split-GAL4 screen and the analysis of the axonal projection patterns of single cells revealed that γ, α′/β′, and α/β Kenyon cells can only be further divided into seven different cell types (Aso Y et al., 2014 *ELife*). The parallel axon fibers of each of the seven types of Kenyon cells occupy specific layers within the γ, α′/β′, and α/β lobes. Two Kenyon cell types divide the γ lobe into the main and dorsal layers, two types divide the α′/β′ lobe into the middle and anterior–posterior layers, and three Kenyon cell types divide the α/β lobe into the posterior, core, and surface layers (Aso Y et al., 2014 *ELife*). We have added a sentence to emphasize the classifications of αβ and γ neuron subsets, and added the related reference in the Discussion section. Page 10, lines 237-239. See below:

“The αβ and γ neurons are classified into αβ core, αβ surface, αβ posterior, γ main, and γ dorsal neuron subsets according to the morphology of their axons [15].”

Minor Comments
1. For non-experts, it would be helpful to include images, or at least diagram showing the mushroom body lobes in Figures 1 and 2. Also please specify the expression pattern near the graph, in addition to the driver line.
Author’s reply:

Thank you for your helpful suggestions. We have performed the imaging experiments and added the GFP expression patterns of mushroom body GAL4 lines that were used in Fig 1 and Fig 2 as suggested (**S1 Fig**; see below).

**S1 Fig. GFP expression pattern driven by MB-GAL4s used in this study.**

(A) The expression pattern of *R13F02-GAL4-*driven GFP expression in αβ, α′β′, and γ neurons. (B) The expression pattern of *R16A06-GAL4-*driven GFP expression in γ neurons. (C) The expression pattern of *5HT1B-GAL4-*driven GFP expression in γ neurons. (D) The expression pattern of *C739-GAL4-*driven GFP expression in αβ neurons. (E) The expression pattern of *VT49246-GAL4-*driven GFP expression in αβ neurons. (F) The expression pattern of *VT30604-GAL4-*driven GFP expression in α′β′ neurons. (G) The expression pattern of *VT57244-GAL4-*driven GFP expression in α′β′ neurons. (H) The expression pattern of *OK107-GAL4-*driven GFP expression in αβ, α′β′, and γ neurons.

Reviewer #3: Lee et al. addressed different subsets of mushroom body neurons for water-rewarded long-term memory (wLTM). The main findings of this work are: 1) post-training protein synthesis and dCREB2b functions in a/b-surface and g-dorsal neurons are essential for wLTM; 2) neurotransmission of the neurons at the wLTM retrieval is necessary for conditioned odor approach; 3) odor responses in these mushroom body neurons undergo training-dependent changes. Overall, results are more or less straightforward, and I am generally supportive for publication if the authors address following points.

1) Discussion is very minimalistic. For example, the Wu lab identified the PAM-b’1 neurons conveying water reward for wLTM (Shyu 2017 Nat Commun), while the current study showed that a’/b’ neurons have limited roles in wLTM. Why? How do the authors suggest the reward input in the b’ lobe causes associative plasticity in the a/b and g neurons?

Author’s reply:

We have added a paragraph in the Discussion section for the possibilities of the memory transfer from α′β′ neurons to γ and αβ neurons. Page 11, lines 256-285. See below:

“Our previous study suggests that PAM-β′1 neurons convey the water-rewarding event as the US signal to the MB β′ lobes, and the neurotransmission in α′β′ neurons is required for wLTM consolidation [1]. How the wLTM is transferred from α′β′ neurons and finally stored in αβ surface and γ dorsal neurons through system consolidation is still unclear. In both shock-punitive and sugar-reward LTMs, the neurotransmission in α′β′, γ, and, αβ neurons is required for at least 3 hours after conditioning, but the expression of 24-hour shock-punitive or sugar-rewarded memories only requires neurotransmission in αβ neurons [28]. In addition, the expression of DopR1, a D1-like dopamine receptor, in the γ neurons is sufficient to fully support the shock-punitive STM and LTM in DopR1 mutant background (*dumb^2^*), suggesting that dopamine-mediated odor/shock association is registered in γ neurons and finally stabilized and maintained in αβ neurons for long-term storage [32]. It might be possible that other MB input neurons (i.e., PAM) convey water rewarding events to MB αβ surface and γ dorsal neurons individually during water drinking in thirsty flies, which allow the initial odor/water associations in αβ surface and γ dorsal neurons [1, 11]. The synaptic output from α′β′ neurons is required for stabilizing and strengthening the associative plasticity in αβ surface and γ dorsal neurons during wLTM consolidation. How the α′β′ neurons communicate with αβ surface and γ dorsal neurons in wLTM consolidation phase, remains unclear. The intrinsic MB neurons, also known as the dorsal paired medial (DPM), arborize widely throughout the MB lobes and play a critical role in modulating MB functions [33-38], including the consolidation of olfactory memories [9, 39, 40]. It might also be possible that DPM neurons receive the input from α′β′ neurons and transmit to αβ surface and γ dorsal neurons during wLTM consolidation. In addition, the output of MB Kenyon cells is conveyed to 34 neurons (MB output neuron, MBON) of 21 cell types per brain hemisphere, and 9 MBONs receive the inputs from α′β′ neurons [41]. Another possibility is that the activity from α′β′ neurons is transmitted to αβ surface and γ dorsal neurons via the relevant α′β′ MBONs and their downstream neurons. Therefore, it is noteworthy to test the physiological roles of DPM neurons and α′β′ MBONs during consolidation phase of wLTM.”

Another point of question is whether post-training protein synthesis is required in the a/b-surface and g-dorsal neurons. This could have been easily addressed with ricin-cs. The authors should provide more discussion.

Author’s reply:

Thank you for your comments. We have performed the experiments and found that blocking *de novo* protein synthesis in αβ surface and γ dorsal neurons disrupted wLTM. We have included the new data in the revised manuscript (**S3 Fig**; see below).

**S3 Fig. Blocking *de novo* protein synthesis in αβ surface and γ dorsal neurons during memory formation disrupts wLTM.** (A) Blocking protein synthesis in αβ surface neurons using *VT20803-GAL4* to drive the expression of activated RICIN^CS^ (30 °C) during memory formation impaired wLTM (left panel). Each value represents mean ± SEM (N = 11). ^*^, p < 0.05; one-way ANOVA followed by Tukey’s test. (B) Blocking protein synthesis in γ dorsal neurons using *R93G04-GAL4* to drive the expression of activated RICIN^CS^ (30 °C) during memory formation impaired wLTM (left panel). Each value represents mean ± SEM (N = 11). ^*^, p < 0.05; one-way ANOVA followed by Tukey’s test.

2) “Engram neurons” and “cellular memory trace” should be used more carefully. These terms refer to cells that receive CS and US information and undergo plastic changes necessary and sufficient for memory (Gerber 2004 Curr Opin Neurobiol; Tonegawa 2015 Neuron). In that sense, the authors’ definition “The cellular memory traces can be any neural activity change induced by learning” (L173-4) is too forgiving. Under this definition, whatever changes downstream of neurons undergoing associative plasiticty can be included. The authors should tone down because these neurons undergoing training-dependent odor responses may not receive the information of water reward during training (cf. Shyu 2017).

Author’s reply:

Thank you for your comments, we have revised all the descriptions of “engram neurons” and “cellular memory trace” in whole manuscript as suggested.

3) Potential effects on water drinking by expression of dCREB2b in Kenyon cells should be experimentally tested because there are several studies showing that these cells are also important in regulation of feeding (e.g. Tsao 2018 eLife; Landayan 2018 Sci Rep).

Author’s reply:

In our previous study, we have identified that thirsty flies showed normal 3-min and 3-hour water-reward memories by adult stage-specific expression of *dCREB2b* transgene in whole mushroom body neurons (see below **Figure R4**; image is adapted from Shyu WH et al., 2017 *Nat. Comm.*). The 3-min and 3-hour water memories were not formed if flies had defects in water-drinking behavior, which suggests that inducible expression of *dCREB2b* in mushroom body neurons does not impair water-drinking in thirsty flies.

**Figure R4. (e)** Adult-stage-specific expression of the *dCREB2b* repressor in MBs abolished 24-h memory, leaving 3-min and 3-h memories intact. Left panel, the R13F02-GAL4 expression pattern (green). The brain was immunostained using DLG antibody (magenta). Scale bar, 50 mm. Each value represents mean ± s.e.m. (N = 8 for each bar). *P < 0.05; ANOVA followed by Tukey’s test. (adapted from Shyu WH et al., 2017 *Nat. Comm.*, Fig 1).

Besides, we have performed the water preference assays in *OK107-GAL4 > UAS-CREB2b; tub-GAL80^ts^* flies. The same inducible protocol for *dCREB2b* expression in adult stage was used in this experiment. Adult flies were raised at 18 °C and then transferred to 30 °C for three days before water preference assays to remove *tub-GAL80ts* inhibition of *GAL4* activity. We found that inducible expression of *dCREB2b* in whole mushroom body neurons using *OK107-GAL4* did not affect water preference (see below **Figure R5**). Taken together, the wLTM defect in the inducible *dCREB2b* expression in mushroom body was not caused by the deficiency of water-drinking or water-seeking behavior in thirsty flies.

**Figure R5.** Inducible expression of *dCREB2b* in mushroom body neurons did not affect water preference in thirsty flies. Each value represents mean ± s.e.m. (N = 8 for each bar). P > 0.05; one-way ANOVA.

4) I do not understand why the authors switched to use shibirets1 to narrow down the KC types (Figure 3, 4). Basically, expressions of shi and dCREB2b/ricin-cs address different cellular processes.

Author’s reply:

In our manuscript Fig 2, we found that adult stage-specific expression of *dCREB2b* in αβ or γ neurons disrupted wLTM. However, our previous studies identified the synaptic output in mushroom body αβ and γ neurons is only required for wLTM retrieval but not for acquisition and consolidation (Shyu WH et al., 2017 *Nat.Comm.*). We therefore were trying to ask whether the synaptic output in small individual αβ and γ neuronal subsets are critical for retrieval of wLTM, and performed the behavioral experiments with *shi^ts^* expressing in αβ and γ neuronal subsets, respectively (Fig 3 & Fig 4). After identifying the synaptic output in small subsets of mushroom body neurons is critical for wLTM retrieval, we then tested whether the CREB activity in the same neuronal subset is necessary for wLTM formation (Fig 5)

5) The title should be more specific. Current title just says that CREB2 is necessary for wLTM in some neural circuits.

Author’s reply:

We have changed the title to “ Mushroom body subsets encode CREB2-dependent water-reward long-term memory in *Drosophila*”.

6) Is there any evidence supporting the claim “wLTM can last for over 24-hours without any decay” (L24)? Shyu 2017 Nat Comm seems to show some, not huge though, decay.

Author’s reply:

In our previous study, we have identified that wLTM lasts to 32-hour and found no significant decline in the memory performance across this time period (see below **Figure R6**; image is adapted from Shyu WH et al., 2017 *Nat. Comm.*). However, over two-thirds of the flies perished at the 32-hour time point. Beyond this point, most of the animals perished, presumably because of dehydration. Therefore, we only examined the time points to 32-hour after odor/water conditioning. We have also modified the original sentence to “wLTM, which can last for over 24 hours without a significant decline”. Page 2, lines 24-25.

**Figure R6. (a)** Olfactory memory curve after water conditioning in wild-type flies. After a single training session, different fly populations were tested once for water memory 0 (3 min), 1, 3, 6, 12, 24 and 32 h after training. Each value represents mean ± s.e.m. (N = 9, 8, 8, 8, 8, 8 and 7 from left to right points). P > 0.05; analysis of variance (ANOVA). (adapted from Shyu WH et al., 2017 *Nat. Comm*., Fig 1).

7) “ANOVA followed by Tukey’s test” (694-5, 719-20). Is it correct? It looks the authors tested the difference from zero (Figure 6B, 7B).

Author’s reply:

Thank you for your helpful comments. We have revised the statistical way accordingly in Fig 6 and Fig 7, and the descriptions for the statistical results were revised to “^*^, p < 0.05; statistically significantly different from zero; one sample t-test.”

**References:**

Shyu WH, Chiu TH, Chiang MH, Cheng YC, Tsai YL, Fu TF, et al. Neural circuits for long-term water-reward memory processing in thirsty Drosophila. ***Nat. Comm.*** 2017;8:15230. Epub 2017/05/16. doi: 10.1038/ncomms15230. PubMed PMID: 28504254; PubMed Central PMCID: PMCPMC5440665.

Chen CC, Wu JK, Lin HW, Pai TP, Fu TF, Wu CL, et al. Visualizing long-term memory formation in two neurons of the Drosophila brain. ***Science***. 2012;335(6069):678-85. Epub 2012/02/11. doi: 10.1126/science.1212735. PubMed PMID: 22323813.

Aso Y, Hattori D, Yu Y, Johnston RM, Iyer NA, Ngo TT, et al. The neuronal architecture of the mushroom body provides a logic for associative learning. ***Elife***. 2014;3. Epub 2014/12/24. doi: 10.7554/eLife.04577. PubMed PMID: 25535793.
